# Survival and prognostic factors in patients with synchronous multiple primary esophageal squamous cell carcinoma receiving definitive radiotherapy: A propensity score-matched analysis

**DOI:** 10.3389/fonc.2023.1132423

**Published:** 2023-03-24

**Authors:** Wenyi Wang, Xiaoxu Liu, Jun Dang, Guang Li

**Affiliations:** ^1^ Department of Radiation Oncology, The First Affiliated Hospital of China Medical University, Shenyang, China; ^2^ Department of Surgical Oncology and General Surgery, The First Affiliated Hospital of China Medical University, Shenyang, China

**Keywords:** esophageal cancer, multiple primary cancer, definitive radiotherapy, survival, nomogram

## Abstract

**Purpose:**

To compare the lesion characteristics and radiotherapy efficacy of patients with single and multiple esophageal squamous cell carcinoma (ESCC), to evaluate the effect of multiple lesions on ESCC, and establish a nomogram survival prediction model for patients with synchronous multiple primary esophageal squamous cell carcinoma (SMPESCC) who received definitive radiotherapy.

**Materials and methods:**

The study enrolled 1,034 patients with ESCC who underwent definitive radiotherapy between 2010 and 2020. The efficacy of radiotherapy was compared between 101 patients with SMPESCC and 933 patients with single ESCC. Propensity score matching was used to control for potential confounders. For patients with SMPESCC, a nomogram prediction model was established based on the Cox regression model.

**Results:**

The median OS was 30.00 (95% CI = 25.08-34.92) months for the single lesion group and 19.00 (95% CI = 15.51-22.48) months for the multiple cancer group respectively. Multivariate COX regression analysis showed that multiple cancer was an independent prognostic factor for ESCC patients (HR=1.89, 95%CI=1.49-2.38, P<0.001). Cox multivariate analysis of SMPESCC patients showed that T stage (P =0.002), chemotherapy (P =0.006), and lesion spacing (P =0.004) were independent prognostic factors associated with OS. The nomogram was established by combining T stage, chemotherapy, and lesion spacing, and Harrell’s C index was 0.711 after internal cross-validation. The calibration curve and decision curve analysis confirmed that the nomogram survival prediction model had a good predictive value for individual survival.

**Conclusions:**

The survival rate of single esophageal cancer is significantly better than that of multiple lesions. Patients with SMPESCC exhibit worse survival than patients with single ESCC. Multiple lesions have a significant impact on the survival of patients with ESCC. The nomogram model established for SMPESCC patients can well predict the individual survival of patients.

## Introduction

Esophageal cancer (EC) is the ninth most common cancer worldwide (1). More than 450000 people worldwide are infected by EC (2). In recent years, more advanced therapies have been used in clinics. This has increased the survival time of EC patients. Meanwhile, the incidence rate of multiple primary cancer developing in patients with EC is increasing, which has been reported in the literature between 5.81% and 32.2% (3–5). At present, the research on EC with multiple primary cancers mainly focuses on EC and other sites, such as head and neck cancer, stomach cancer, etc. However, the different locations in the esophagus are rarely studied. Multiple primary esophageal carcinoma is defined as two or more primary malignant tumors occurring simultaneously or successively in different parts of the esophagus. The second cancer occurring within 6 months is defined as simultaneous multiple primary esophageal carcinoma (SMPEC), and the second cancer occurring over 6 months is referred to as metachronous multiple primary esophageal carcinomas. Some studies have shown that smoking and drinking are closely related to the occurrence of the second primary cancer of EC (6, 7), but the risk factors for SMPEC remain unclear. At present, the reported 5-year survival rate of single EC is mostly between 15%-25% (8–10). However, there are few studies on the survival rate of SMPEC, and the results of existing studies vary widely, ranging from 7.7% to 28.9% (11, 12). The impact of multiple lesions on the prognosis of EC has not been determined. In addition, there is no standard treatment strategy for patients with SMPEC. Currently, radical treatment is mostly used to improve the survival rate.

Therefore, this study analyzed the characteristics and prognosis of synchronous multiple primary esophageal squamous cell carcinoma (SMPESCC) patients who received definitive radiotherapy in a single institution within 10 years, aiming to determine the risk factors for its occurrence and the impact of multiple lesions on the survival of EC, and to evaluate the efficacy and prognostic factors of radiotherapy, to provide a reference for clinical doctor’s treatment decisions.

## Materials and methods

### Patients

A total of 1034 patients with ESCC from January 2010 to June 2020 received definitive radiotherapy at the Department of Radiotherapy of the First Affiliated Hospital of China Medical University were recruited, resulting in 101 patients with SMPESCC, accounting for about 9.77% of the total. The diagnosis of multiple primary carcinomas is based on criteria proposed by Warren et al. in 1933 (13): (1) the tumors must be malignant on histologic examination; (2) the tumors must be separated by normal mucosa; and (3) the possibility that the second tumor is metastatic must be excluded. In addition, all lesions met the radiographic diagnostic criteria proposed by Xiao et al. (14).

The exclusion criteria were: (1) Having received surgery; (2) Accompanied by hypopharyngeal carcinoma or other local cancers; (3) The pathological type was adenocarcinoma or other types. The stage and location of a lesion of EC is defined according to the American Joint Commission on Cancer (AJCC) Tumor Node Metastasis (TNM) Classification of the esophageal tumor (8th Edition, 2017). T-stage and N-stage were determined by endoscopic ultrasound and chest enhanced CT. The length of the affected lesions was determined according to the length measured by gastrointestinal endoscopy before treatment. For esophageal stenosis, gastrointestinal barium X-ray imaging or chest enhanced CT was used to determine the length. Cervical lymph node involvement was determined by chest enhanced CT and cervical Doppler ultrasound.

This study was approved by Institutional Review Board of The First Hospital of China Medical University (AF-SOP-07-1.1-01), and all patients were enrolled in our department after written informed consent was obtained.

### Treatment

All patients were treated with definitive radiotherapy and received the usual segmentation regimen: 1.8-2.5Gy each time and 5 times per week with a 6-mV linear accelerator. The radiotherapy dose was 50-66Gy/25-33fractions. Among them, 9 patients received three-dimensional conformal radiotherapy, and 92 patients received intensity-modulated radiotherapy. Fourteen of them discontinued/did not complete planned radiotherapy. 67 patients received synchronous/sequential chemotherapy, mainly 2-4 cycles of platinum.

### Follow up

Overall survival (OS) was calculated from the date of the first diagnosis to the point of death or last follow-up. Patients were followed up until March 1, 2022, mainly by outpatient review and telephone. Patients who were lost to follow-up or survived at the last follow-up were recorded as censored data.

### PSM

PSM is performed by the “MatchIt” package in R software (version 4.1.3). The general baseline data of the single and multiple lesion groups, including sex, age, lymph node metastases, history of alcohol and tobacco, chemotherapy and radiotherapy dose, were matched using a 1:1 nearest neighbor method, with a caliper value set to 0.05. Mean differences and equilibrium between all baseline covariates in exposed and control groups were assessed before and after matching to assess the degree of balance, with a 50% increase in equilibrium for a given covariable indicating good propensity score performance.

### Statistical analysis

Statistical analysis was performed using SPSS 26.0 (Chicago, IL). A Chi-square test was used to compare the distribution of clinicopathological data in each group. Risk factors for multiple esophageal squamous cell carcinoma were determined by Logistic regression analysis. Cox regression analysis was used to evaluate whether multiple lesions were independent prognostic factors for esophageal squamous cell carcinoma patients who received definitive radiotherapy. Kaplan-Meier method and log-rank test were used to draw the survival curve. Univariate and multivariate Cox proportional risk regression models were used to assess prognostic factors in patients with SMPESCC undergoing definitive radiotherapy. All tests were two-sided, and P values less than 0.05 were considered statistically significant.

Based on the Cox regression model, the nomogram of OS-related prognostic factors in SMPESCC patients was established. Discrimination and calibration were used to evaluate the performance of the prognostic model. The Discrimination of the nomogram was measured by Harrell’s Consistency Index (C-index) and cross-validated with 1000 bootstrap samples to prevent overfitting of the model. The c-index ranges from 0.5 to 1, and a higher C-index indicated a more discriminative nomogram model. Calibration was generally assessed by plotting the predicted probability of survival (horizontal axis) against the actual probability of survival (vertical axis) from the nomogram model. The perfect and accurate calibration curve showed that all observation points fell on the diagonal line of 45 degrees. Decision curve analysis (DCA) was performed to determine the clinical net benefit of model reliability at different probability thresholds.

## Results

### Clinical characteristic

Of the 101 SMPESCC patients, the vast majority (93.07%) were male, aged 45-82 (median: 60 years). 96 patients had two lesions and 5 had three. Among the patients with double primary lesions, most patients (41/96) were synchronous middle thoracic segment and the lower thoracic segment. Among 5 patients with three primary esophageal carcinoma, 2 lesions were located in the lower thoracic segment, and the remaining 1 lesion was located in the neck segment (1/5) and the middle thoracic segment (4/5). The largest T stage in multiple lesions was considered as the total T stage of the patient. T3 stage (39.60%) and T4 stage (35.64%) accounted for the majority of patients. Cervical or supraclavicular lymph node metastasis was observed in 52 patients (51.49%), and mediastinal lymph node metastasis in 68 patients (67.33%). Compared with single esophageal cancer, the risk factors of multiple esophageal cancer were age (P=0.017) and weight loss (P=0.017).

### Impact of multiple lesions on survival of ESCC

**Figure 1:**
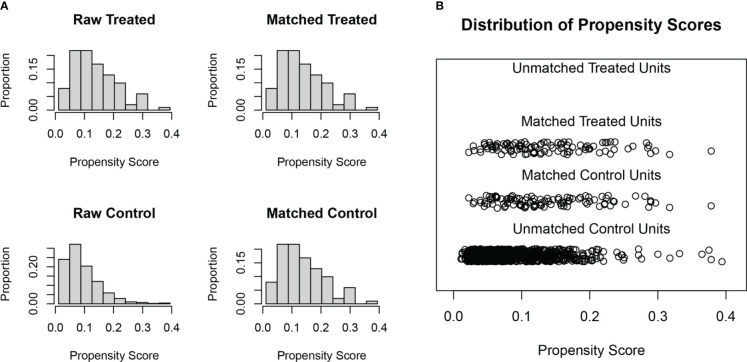
**(A)** Histogram of patients distribution before and after propensity score matching. **(B)** Distribution results of patients after propensity score matching.

**Figure 2:**
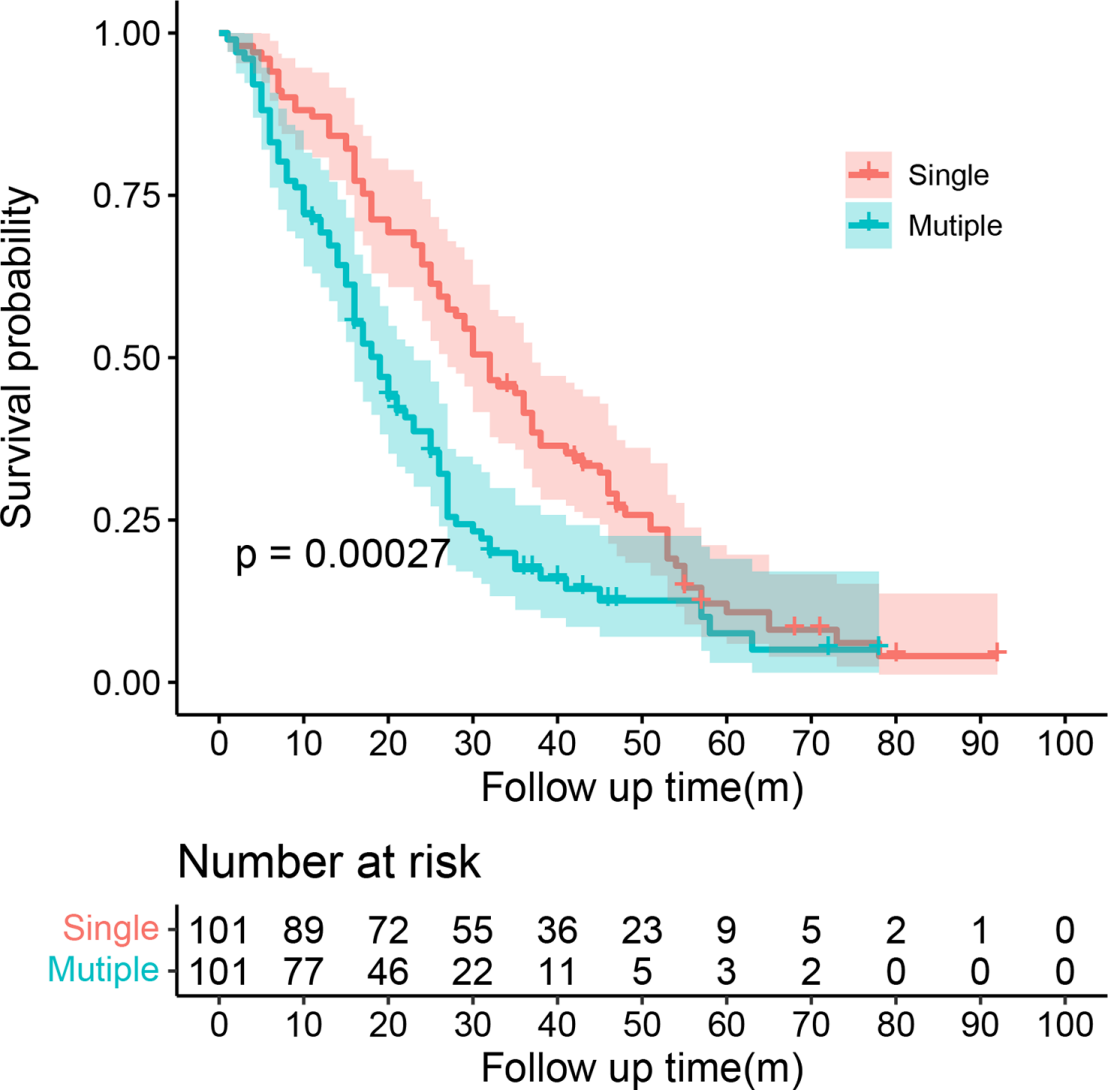
K-M survival curve between the single EC lesion group and multiple EC lesion group.

SMPESCC patients were matched with single lesion ESCC patients undergoing definitive radiotherapy at the same time. The distribution of patients before and after propensity score matching is shown in **Figure 1A**. Finally, 101 patients with isolated ESCC lesions and 101 patients with multiple ESCC lesions were matched (**Figure 1B**, **Table 1**). The equilibrium test showed that the mean difference of the overall distance between the two groups decreased from 0.5897 to 0.0007, and equilibrium increased by 99.78%. The equilibrium of all variables increased by more than 50%, indicating a good matching effect. The median OS was 30.00 (95% CI = 25.08-34.92) months for the single lesion group and 19.00 (95% CI = 15.51-22.48) months for the multiple lesions group respectively. **Figure 2** shows the K-M survival curve between the two groups. Patients with multiple lesions had a significantly shorter survival time than those with single lesions (P<0.001). Multivariate COX regression analysis showed that multiple cancer was an independent prognostic factor for ESCC patients (HR=1.89, 95%CI=1.49-2.38, P<0.001). Other prognostic factors for ESCC patients were T stage (P<0.001) and chemotherapy (P<0.001) (**Table 2**).

**Table 1:** Baseline characteristics before and after propensity score matching.

Characteristics	Before matching			After matching		
	Single lesion (n=933)	Muiti-lesion (n=101)	SMD	Single lesion (n=101)	Multi-lesion (n=101)	SMD
**Sex (%)**			0.174			0.078
Male	827 (88.6)	94 (93.1)		92 (91.1)	94 (93.1)	
Female	106 (11.4)	7 (6.9)		9 (8.9)	7 (6.9)	
**Age, years (%)**			0.265			<0.001
40-50	58 (6.2)	4 (4.0)		9 (8.9)	4 (4.0)	
50-60	268 (28.7)	45 (44.6)		35 (34.7)	45 (44.6)	
60-70	368 (39.4)	36 (35.6)		41 (40.6)	36 (35.6)	
≥70	239 (25.6)	16 (15.8)		16 (15.8)	16 (15.8)	
**T (%)**			0.266			0.097
T1	11 (1.2)	2 (2.0)		1 (1.0)	2 (2.0)	
T2	120 (12.9)	23 (22.8)		17 (16.8)	23 (22.8)	
T3	375 (40.2)	40 (39.6)		47 (46.5)	40 (39.6)	
T4	427 (45.8)	36 (35.6)		36 (35.6)	36 (35.6)	
**N (%)**			0.335			0.077
N0	227 (24.3)	13 (12.9)		11 (10.9)	13 (12.9)	
N1	287 (30.8)	26 (25.7)		27 (26.7)	26 (25.7)	
N2	292 (31.3)	46 (45.5)		43 (42.6)	46 (45.5)	
N3	127 (13.6)	16 (15.8)		20 (19.8)	16 (15.8)	
**Length (%)**			0.212			0.033
<5 cm	404 (43.3)	33 (32.7)		31 (30.7)	33 (32.7)	
5-10cm	466 (49.9)	59 (58.4)		61 (60.4)	59 (58.4)	
≥10 cm	63 (6.8)	9 (8.9)		9 (8.9)	9 (8.9)	
**Smoke (%)**			0.114			0.202
Yes	716 (76.7)	82 (81.2)		90 (89.1)	82 (81.2)	
No	217 (23.3)	19 (18.8)		11 (10.9)	19 (18.8)	
**Drink (%)**			0.225			0.073
Yes	654 (70.1)	80 (79.2)		83 (82.2)	80 (79.2)	
No	279 (29.9)	21 (20.8)		18 (17.8)	21 (20.8)	
**Weight loss (%)**			0.233			0.060
Yes	495 (53.1)	42 (41.6)		45 (44.6)	42 (41.6)	
No	438 (46.9)	59 (58.4)		56 (55.4)	59 (58.4)	
**Chemotherapy (%)**			0.408			0.063
Yes	439 (47.1)	67 (66.3)		70 (69.3)	67 (66.3)	
No	494 (52.9)	34 (33.7)		31 (30.7)	34 (33.7)	
**Radiotherapy**			0.027			0.022
<60Gy	270 (28.9)	28 (27.7)		74 (73.3)	28 (27.7)	
≥60Gy	663 (71.1)	73 (72.3)		27 (26.7)	73 (72.3)	

**Table 2:** Cox univariate and multivariate regression analysis for overall survival of ESCC patients.

Variable	Modality	N (%)	Univariate		Multivariate	
			HR (95%CI)	P-Value	HR (95%CI)	P-Value
Lesion	Single	933 (90.2)	1	<0.001	1	<0.001
	Multiple	101 (9.8)	1.625 (1.299-2.032)		1.885 (1.492-2.381)	
Sex	Male	921 (89.1)	1	0.353	1	0.682
	Female	113 (10.9)	0.903 (0.729-1.120)		0.949 (0.740-1.218)	
Age	40-50	62 (6.0)	1	0.019	1	0.097
	50-60	313 (30.3)	1.360 (1.006-1.837)		1.309 (0.965-1.776)	
	60-70	404 (39.1)	1.122 (0.834-1.510)		1.195 (0.886-1.613)	
	≥70	255 (24.7)	1.357 (0.999-1.845)		1.408 (1.030-1.925)	
T stage	T1	13 (1.3)	1	<0.001	1	<0.001
	T2	143 (13.8)	1.239 (0.667-2.301)		1.529 (0.814-2.872)	
	T3	415 (40.1)	1.583 (0.868-2.886)		2.019 (1.092-3.732)	
	T4	463 (44.8)	1.902 (1.045-3.465)		2.353 (1.275-4.344)	
N stage	N0	240 (23.2)	1	0.137	1	0.280
	N1	313 (30.3)	1.144 (0.955-1.371)		1.142 (0.949-1.373)	
	N2	338 (32.7)	1.194 (0.998-1.428)		1.137 (0.944-1.368)	
	N3	143 (13.8)	1.270 (1.015-1.589)		1.242 (0.986-1.565)	
Total lengh	0-5	437 (42.3)	1	0.044	1	0.257
	5-10	525 (50.8)	1.119 (0.977-1.283)		1.069 (0.930-1.229)	
	≥10	72 (7.0)	1.358 (1.045-1.764)		1.238 (0.949-1.614)	
Smoke	No	236 (22.8)	1	0.310	1	0.340
	Yes	798 (77.2)	1.085 (0.927-1.269)		1.095 (0.909-1.319)	
Drink	No	300 (29.0)	1	0.164	1	0.608
	Yes	734 (71.0)	1.108 (0.959-1.279)		1.046 (0.881-1.243)	
Weight loss	No	497 (48.1)	1	0.433	1	0.775
	Yes	537 (51.9)	1.054 (0.924-1.201)		1.020 (0.892-1.166)	
Chemotherapy	No	528 (51.1)	1	<0.001	1	<0.001
	Yes	506 (48.9)	0.751 (0.659-0.857)		0.730 (0.636-0.839)	

### Prognostic analysis of SMPESCC patients

**Figure 3:**
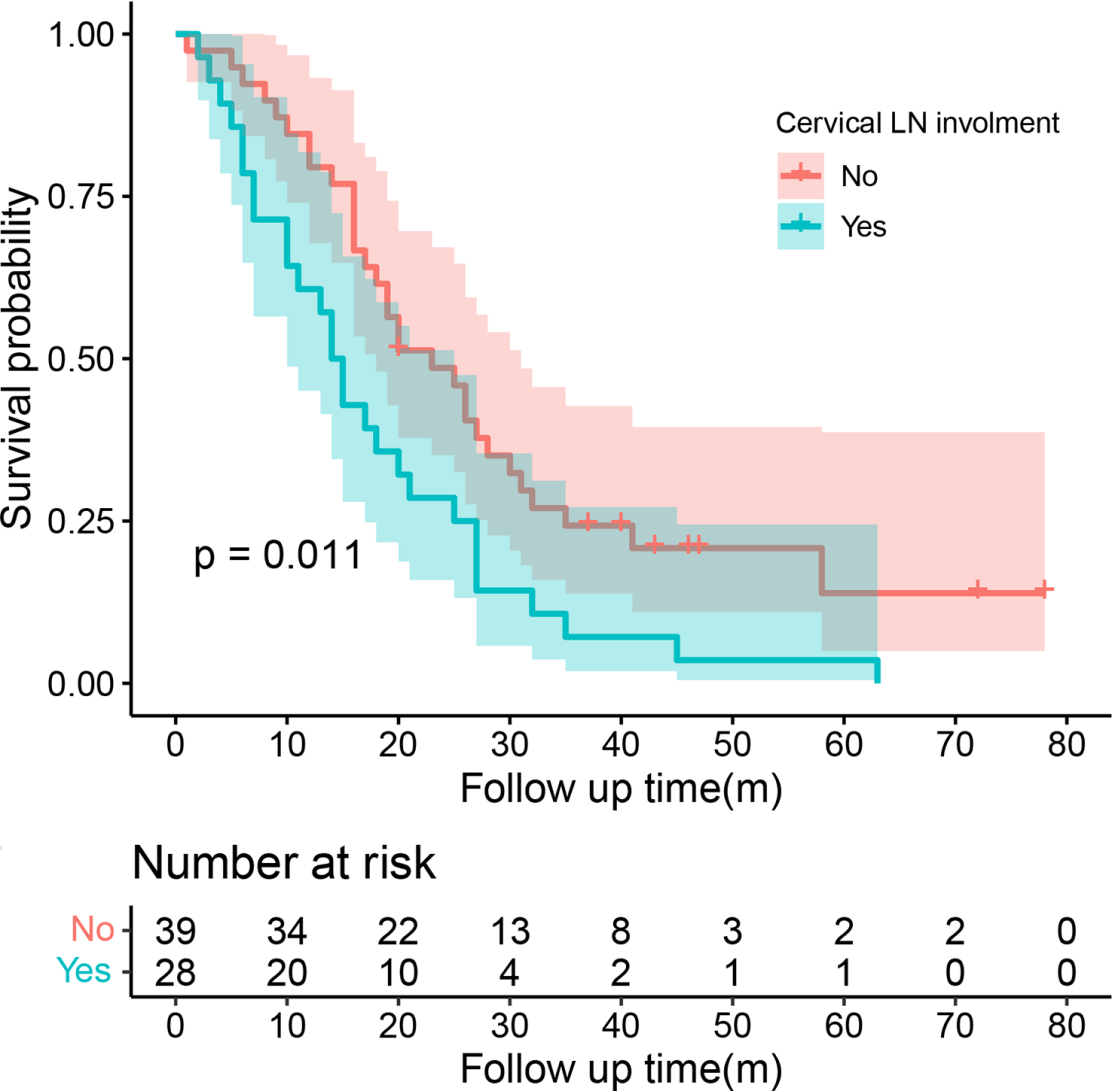
K-M survival curve between the group with or without cervical lymph node involvement.

Univariate COX regression analysis showed that T stage (P=0.008), the distance between two lesions (HR=1.14, 95%CI=1.06-1.22, P<0.001), and chemotherapy (HR=0.64, 95%CI=0.41-0.99, P=0.048) may affect the survival of patients with SMPESCC (**Table 3**). Multivariate regression analysis showed the above factors were also independent prognostic factors for survival of SMPESCC patients, while N stage had no significant relationship with survival. However, when all the lesions of SMPESCC patients were located in the middle or lower thoracic segment, we found that the survival time of patients with cervical lymph node metastasis was shorter than that of those without lymph node metastasis. (**Figure 3**)

**Table 3:** Cox univariate and multivariate regression analysis for overall survival of SPESCC patients.

Variable	Modality	N (%)	Univariate		Multivariate	
			HR (95%CI)	P-Value	HR (95%CI)	P-Value
Sex	Male	94 (93.1)	1	0.178	1	0.497
	Female	7 (6.9)	0.500 (0.182-1.372)		0.676 (0.218-2.096)	
Age	40-50	4 (4.0)	1	0.649	1	0.818
	50-60	45 (44.6)	0.852 (0.262-2.770)		1.022 (0.262-3.989)	
	60-70	36 (35.6)	1.014 (0.310-3.315)		1.229 (0.331-4.557)	
	≥70	16 (15.8)	0.668 (0.188-2.378)		0.900 (0.217-3.727)	
T stage	T1	2 (2.0)	1	0.008	1	0.002
	T2	23 (22.8)	1.657 (0.361-7.600)		4.877 (0.777-30.624)	
	T3	40 (39.6)	3.492 (0.801-15.228)		7.958 (1.389-45.604)	
	T4	36 (35.6)	4.446 (1.002-19.724)		13.480 (2.220-81.832)	
N stage	N0	13 (12.9)	1	0.233	1	0.438
	N1	26 (25.7)	0.770 (0.368-1.610)		0.889 (0.369-2.138)	
	N2	46 (45.5)	1.057 (0.540-2.069)		1.061 (0.468-2.404)	
	N3	16 (15.8)	1.569 (0.718-3.429)		1.696 (0.649-4.432)	
Total length	0-5	33 (32.7)	1	0.083	1	0.864
	5-10	59 (58.4)	1.452 (0.895-2.357)		1.019 (0.566-1.836)	
	≥10	9 (8.9)	2.329 (1.073-5.055)		1.265 (0.506-3.162)	
Smoke	No	82 (81.2)	1	0.599	1	0.610
	Yes	19 (18.8)	0.867 (0.508-1.478)		0.857 (0.475-1.548)	
Drink	No	80 (79.2)	1	0.089	1	0.155
	Yes	21 (20.8)	1.629 (0.928-2.859)		1.663 (0.825-3.354)	
Weight loss	No	42 (41.6)	1	0.337	1	0.950
	Yes	59 (58.4)	1.238 (0.801-1.912)		1.018 (0.591-1.751)	
Chemotherapy	No	67 (66.3)	1	0.048	1	0.006
	Yes	34 (33.7)	0.637 (0.407-0.997)		0.456 (0.262-0.794)	
Distance *	–	–	1.136 (1.057-1.220)	<0.001	1.156 (1.047-1.277)	0.004

* Distance: The length between the centers of lesions. - : Distance is a continuous variable

**Figure 4:**
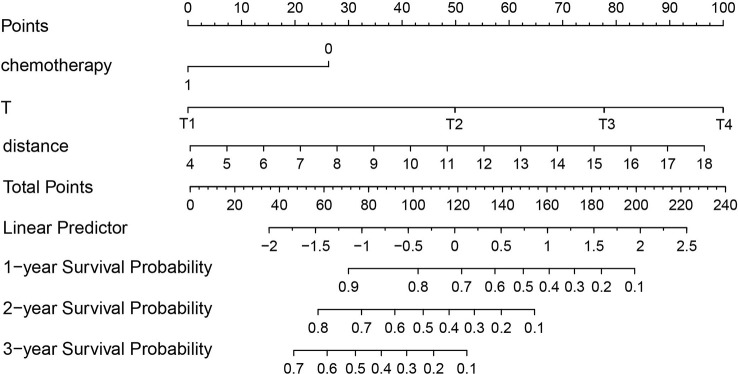
Nomogram for predicting SMPESCC patient survival.

**Figure 5:**
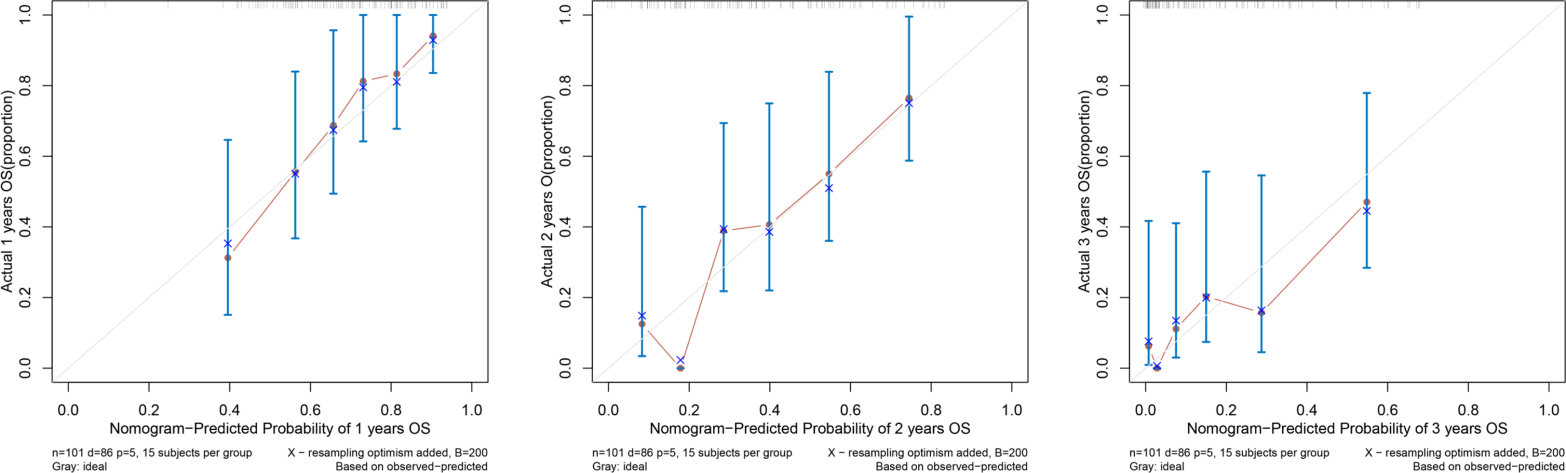
Calibration curve for predicting 1-, 2-, and 3-year OS.

**Figure 6:**
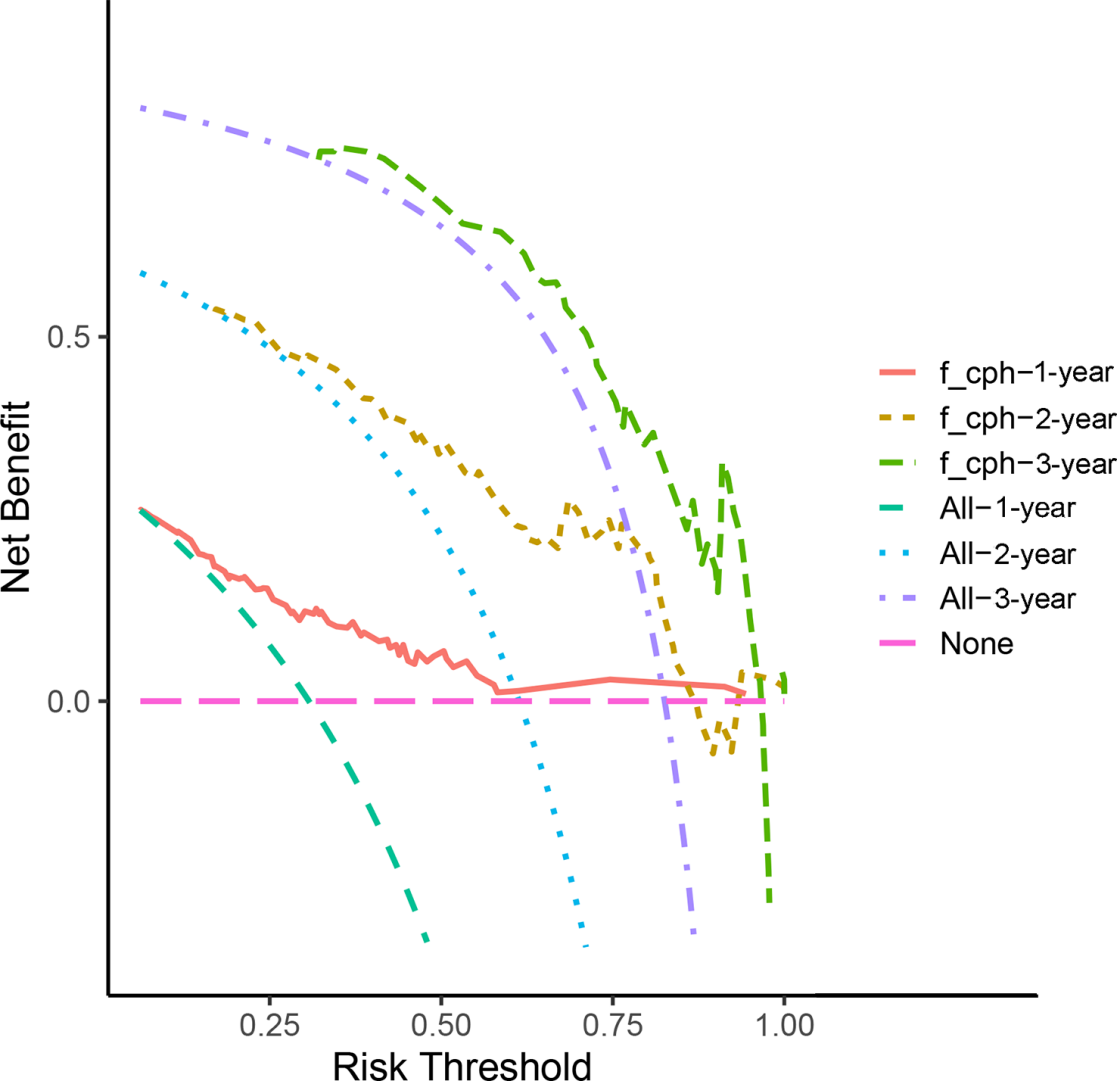
Decision curve analysis demonstrated positive net benefits by the nomogram model. The “f_cph-1,2,3-year” lines represent the net benefit of nomogram model, the “All-1,2,3-year” lines and “None” line represent that no patients and all patients would die, respectively.

According to the results of the Cox regression model, the prognostic nomogram of OS was established by integrating the independent factors of OS. Nomogram according to T stage, the distance between two lesions, and the distribution point of chemotherapy (**Figure 4**). The axes of the individual covariates in the nomogram are plotted by ranking the effect estimates, with each covariate assigned a score on the scale. The total score for each covariate accumulation corresponded to the predicted probability of 1-year (AUC=0.764), 2-year (AUC=0.795), and 3-year survival (AUC=0.848). The bootstrap method was used to randomly select samples from the original data set. After 1000 repetitions, Harrell’s C index was 0.711, indicating the good performance of the model. The calibration curves for the predicted survival probability and the observed probability were close to the 45° diagonal (**Figure 5**). DCA was performed to determine the reliability of the prediction results based on nomograms under different decision thresholds. The DCA shows the positive net benefits of the nomogram model. Decision curves predicting the survival of patients with SMPESCC after 1 year, 2 years, and 3 years are shown in **Figure 6**.

## Discussion

As the survival rate of patients with EC improves, the second primary cancer will become an increasing concern for patients with EC. The pathogenesis of multiple primary EC remains unclear. Strong et al. (15) proposed the field cancerization hypothesis that the whole esophagus was the entirety of the cancerization and the regional tissues were in different stages of the cancerization. When various pathogenic factors accumulate to a certain stage, one or more cancer foci would appear successively or simultaneously in different parts of the esophagus. Kuwabara et al. (16) performed a genetic analysis of SMPEC and showed that SMPEC patients presented with alterations in one or more gene loci. The most commonly mutated genes were TP53 and D18S61, and there were great differences in gene mutations between different patients. In our study, the incidence of SMPESCC was 9.77%, and the majority of SMPESCC patients were male. Gender differences were also observed in patients with EC, for reasons that are unclear (17). Yingcai H et al. suggested that up-regulation of Y-BOX9 in the sex-determining region was associated with the development of ESCC (18). However, we did not find that EC patients of any gender were more likely to develop multiple cancer. It was reported that individuals diagnosed at 60–79 years old, with earlier stage and/or moderately differentiated EC were more likely to get EC-multiple cancer (3). In addition to the influence of internal factors, long-term and chronic stimulation of external carcinogenic factors such as tobacco and ethanol can lead to regional carcinogenesis of the upper airway and digestive tract (19, 20). Multiple studies had shown that long-term smoking and alcohol consumption were also associated with the emergence of multiple cancer (6, 21). In our study, smoking and alcohol consumption were not significantly associated with SMPESCC, while older patients and those with previous weight loss were more likely to develop SMPESCC.

The survival of multiple primary cancers varies greatly in different pieces of literature because it is affected by the location of the tumor, the time of tumor occurrence, and the tumor stage. A large study of patients with multiple primary cancer showed significantly better survival than patients with a single primary cancer (22). Possible explanations for this were that different patient populations had different tumor sites (mainly favorable sites) and/or disease stages (mainly early stages). For patients with SMPESCC, Yang et al. (23) suggested that there was no significant difference in survival between SMPESCC and single ESCC in the study of SMPESCC patients undergoing surgery. However, they also proposed that when the T stage was T3 or T4, the survival time of SMPESCC was shorter. In our study, after balancing baseline characteristics between the single and multiple groups, we found that ESCC with multiple lesions was significantly associated with poorer overall survival. Consistent with the above studies, most of patients in our study had high T stage. Furthermore, our study showed that multiple primary cancer could be an independent prognostic factor for survival of ESCC patients receiving definitive radiotherapy, which was consistent with the conclusions of Yoshifumi B et al. (6).

In a study of simultaneous multiple gastric cancers, the results showed that the primary and secondary lesions were located in the same vertical direction, and the size of the two lesions was positively and significantly correlated (24). In our study, most of the major lesions in SMPESCC patients were located in the middle thoracic segment (34.65%) and lower thoracic segment (51.49%), and the minor lesions were mainly located in the lower thoracic segment (60.40%). However, no significant correlation was observed between the vertical position of the two lesions (P=0.053). For patients with inoperable EC, the clinical staging was mostly used to evaluate the tumor status. According to the degree of CT invasion and the relationship between the lesions and surrounding tissues and organs, these SMPESCC patients performed clinical T staging. Most patients were in T3 stage and T4 stage. Univariate and multivariate analyses showed that patients with early T stage had a higher survival rate than those with advanced T stage, indicating that this clinical T stage can be used as an independent prognostic factor for SMPESCC patients receiving definitive radiotherapy. No significant correlation was observed between N stage and prognosis. But it does not mean that lymph node involvement has no effect on survival. We found that cervical lymph node involvement was indicative of a poor prognosis in SMPESCC patients whose lesions were located in the mid-chest or lower-chest segments. The effect of tumor length on the prognosis of EC had been controversial. For SMPEC patients, the study by Eloubeidi. M.A et al. pointed out that the shorter the tumor length, the longer the survival time (25). However, our results showed no significant association between total tumor length and survival, which means that more studies were needed to confirm the relationship in the future. We used the center of the lesion to judge the location of the lesion and included the spacing of the lesion in the multivariate regression model. The results showed that the distance between lesions significantly affected the survival time of patients with SMPESCC. The longer the distance, the shorter the survival time of patients. In inoperable locally advanced EC, concurrent chemoradiotherapy had been shown to significantly improve survival compared with radiotherapy alone (26). Multivariate analysis of this study showed that chemotherapy was an independent prognostic factor for SMPESCC patients who received definitive radiotherapy (P=0.006), suggesting that SMPESCC patients would also benefit from chemotherapy.

Nomograms can generate individual probabilities of clinical events by integrating different prognostic variables, thus fulfilling our need for clinical integration models. To more clearly demonstrate the ability of each variable to predict the survival of patients with SMPESCC, we constructed a nomogram based on the Cox regression model. The nomogram is based on tumor T stage, tumor spacing, and chemotherapy mapping. Internal cross-validation showed that the prediction model had a good prediction ability for OS (C-index=0.711). At the same time, the calibration curves for the predicted probabilities and the actual observed events of the 1-year, 2-year, and 3-year OS were close to a 45-degree diagonal. DCA also showed a positive net benefit, indicating a good calibration and clinical benefit of our model.

## Limitation

This study is a retrospective article. Although multiple esophageal cancer is still a rare disease, we did not have enough cases to explore the prognosis, so there was no external verification after the nomogram was developed. Therefore, prospective studies with a larger sample size are needed to further elucidate the prognostic factors of SMPESCC patients and external validation of nomograms.

## Conclusion

In conclusion, SMPESCC is a relatively rare but aggressive tumor. The appearance of multiple primary cancer makes the prognosis of esophageal cancer patients more pessimistic. Multivariate analysis showed that T stage, lesion spacing, and chemotherapy were independent prognostic factors, suggesting that SMPESCC patients who received definitive radiotherapy could benefit from chemotherapy. Based on the independent prognostic factors associated with OS, we established Nomogram models to predict 1-year, 2-year, and 3-year survival of SMPESCC patients, which can effectively predict individual survival time. In the meantime, our results need to be validated by prospective large clinical studies.

## Data availability statement

The raw data supporting the conclusions of this article will be made available by the authors, without undue reservation.

## Ethics statement

The studies involving human participants were reviewed and approved by Medical Research Ethics Committee of the First Affiliated Hospital of China Medical University. The patients/participants provided their written informed consent to participate in this study.

## Author contributions

WW: Conceptualization, Methodology, Formal analysis, writing-Original Draft; XL: Data Curation, Visualization; JD: Writing-Review & Editing. GL: Validation, Supervision, Writing-Review & Editing. All authors contributed to the article and approved the submitted version.
